# Candidate Heterotaxy Gene FGFR4 Is Essential for Patterning of the Left-Right Organizer in Xenopus

**DOI:** 10.3389/fphys.2018.01705

**Published:** 2018-12-04

**Authors:** Emily Sempou, Osaamah Ali Lakhani, Sarah Amalraj, Mustafa K. Khokha

**Affiliations:** Department of Pediatrics, Yale School of Medicine, Yale University, New Haven, CT, United States

**Keywords:** FGF signaling, left-right patterning, gastrulation, Xenopus, congenital heart disease, heterotaxy

## Abstract

Congenital heart disease (CHD) is the most common birth defect, yet its genetic causes continue to be obscure. Fibroblast growth factor receptor 4 (*FGFR4*) recently emerged in a large patient exome sequencing study as a candidate disease gene for CHD and specifically heterotaxy. In heterotaxy, patterning of the left-right (LR) body axis is compromised, frequently leading to defects in the heart's LR architecture and severe CHD. FGF ligands like FGF8 and FGF4 have been previously implicated in LR development with roles ranging from formation of the laterality organ [LR organizer (LRO)] to the transfer of asymmetry from the embryonic midline to the lateral plate mesoderm (LPM). However, much less is known about which FGF receptors (FGFRs) play a role in laterality. Here, we show that the candidate heterotaxy gene *FGFR4* is essential for proper organ situs in *Xenopus* and that frogs depleted of fgfr4 display inverted cardiac and gut looping. *Fgfr4* knockdown causes mispatterning of the LRO even before cilia on its surface initiate symmetry-breaking fluid flow, indicating a role in the earliest stages of LR development. Specifically, *fgfr4* acts during gastrulation to pattern the paraxial mesoderm, which gives rise to the lateral pre-somitic portion of the LRO. Upon *fgfr4* knockdown, the paraxial mesoderm is mispatterned in the gastrula and LRO, and crucial genes for symmetry breakage, like *coco, xnr1*, and *gdf3* are subsequently absent from the lateral portions of the organizer. In summary, our data indicate that FGF signaling in mesodermal LRO progenitors defines cell fates essential for subsequent LR patterning.

## Introduction

Left-right (LR) asymmetry is a major characteristic of the vertebrate body plan. While externally symmetric, chordates display a specific internal LR arrangement of their visceral organs. In patients with heterotaxy, LR development is defective and organs are mispatterned relative to the LR axis, which often results in compromised LR architecture of the heart and clinically severe cardiac dysfunction. Although heterotaxy is a predominantly genetic disease, causal genes remain largely unidentified (Zaidi and Brueckner, 2017). Exome sequencing of congenital heart disease (CHD) patients identified three individuals with damaging mutations in fibroblast growth factor receptor 4 (*FGFR4*). The first patient, with hypoplastic left heart syndrome, had a *de novo* mutation (Asp297Asn). The second patient, with an L-transposition of the great arteries and tricuspid atresia, had an inherited stopgain at Gly705. And finally, a third patient had an inherited damaging splice mutation associated with a hypoplastic main pulmonary artery and a left superior vena cava that emptied into the coronary sinus (Zaidi et al., 2013; Jin et al., 2017). The patients' phenotypes suggest defects in LR patterning consistent with an established role for FGF signaling in LR patterning. We sought to investigate this further by studying the role of FGFR4 in the LR patterning cascade.

FGF signaling is governed by an array of FGF ligands and their receptors (FGFRs). In mammals, 18 FGFs and four FGFRs (FGFR1-4) are employed in a broad variety of developmental processes, including LR patterning (Teven et al., 2014). LR patterning begins at the left-right organizer (LRO) which forms in the posterior mesoderm at the end of gastrulation. At the LRO, cilia beat to create leftward extracellular fluid flow that breaks bilateral symmetry (Essner et al., 2002; Schweickert et al., 2007; Babu and Roy, 2013; Yoshiba and Hamada, 2014). For LR patterning to occur properly, LRO progenitor cells must be specified in the mesoderm, and the LRO has to be physically formed and properly engage in ciliogenesis and cilia signaling. Once the flow signal is detected in the left margin of the LRO, it is transmitted via nodal signaling to the left lateral plate mesoderm (LPM), inducing asymmetric pitx2c expression, which then results in asymmetric organogenesis (Logan et al., 1998; Piedra et al., 1998; Ryan et al., 1998; Yoshioka et al., 1998; Campione et al., 1999; Kawasumi et al., 2011). FGF signaling plays a role in several steps in this process.

Zebrafish homozygous for a stopgain mutation of FGF8 have been shown to physically lack a LRO (Albertson and Yelick, 2005). On the other hand, FGF8 acts differentially in chick, mouse, and rabbit to regulate asymmetric gene expression at the LRO and LPM (Boettger et al., 1999; Meyers and Martin, 1999; Fisher et al., 2002). In contrast, FGF4, stimulates ciliogenesis in the zebrafish LRO, but is not required to physically form the organizer (Yamauchi et al., 2009). Therefore, FGF ligands may act at different steps in the LR cascade; however, the FGF receptors (FGFR) that convey these effects are less well-understood. In zebrafish, FGFR1 is required to establish proper cilia length in several ciliated organs, including the LRO, confirming a role for FGF signals in ciliogenesis (Neugebauer et al., 2009). However, whether any of the other FGFRs play a role in earlier LR patterning steps or ciliogenesis is unknown.

Here, we show in *Xenopus* that *fgfr4* is required during gastrulation to differentiate the paraxial mesoderm that gives rise to the lateral gastrocoel roof plate (GRP), which is the amphibian LRO. F0 CRISPR mediated knockdown of *fgfr4* results in failure to specify the paraxial pre-somitic LRO, resulting in a smaller, mispatterned GRP. Consistently, *fgfr4* depletion leads to inverse LR heart architecture (L-loop) as well as abnormal LR patterning of the LPM, recapitulating the patient heterotaxy phenotype. Altogether, our results indicate that the heterotaxy candidate gene *FGFR4* acts early in gastrulation to specify pre-somitic tissue that is crucial to LRO function and correct organ situs.

## Materials and Methods

### Xenopus

*Xenopus tropicalis* were housed and cared for in our aquatics facility according to established protocols that were approved by the Yale IRB—Institutional Animal Care and Use Committee (IACUC). Embryos were produced by *in vitro* fertilization and raised to appropriate stages in 1/9X MR as per standard protocol (del Viso and Khokha, 2012).

### CRISPR Injection and Validation

Injections of *Xenopus* embryos were carried out at the one-cell stage using a fine glass needle and Picospritzer system, as previously described (del Viso and Khokha, 2012). Small guide RNAs (sgRNAs) containing the following *fgfr4* target sites were designed from the v7.1 model of the *X. tropicalis* genome: CRISPR-1 (exon 3): 5′-AGGAACGTTTGCTGCCGGGAGGG-3′, CRISPR-2 (exon 6): 5′-AGTGTGGTTCCATCAGACCGTGG-3′, and CRISPR-3 (in exon 8): 5′-TGCAGGGGAATACACATGTCTGG-3′. One-cell embryos were injected with 1.5 ng Cas9 Protein (PNA-Bio) and 400 pg of targeting sgRNA and raised to desired stages as previously described in detail (Bhattacharya et al., 2015). For genotyping, F0 embryos were raised to stage 45 and lyzed in 50 mM NaOH as previously described (Bhattacharya et al., 2015). Editing by CRISPR-1 was verified by amplifying from tadpole genomic DNA an 800 bp fragment around the prospective cut site in *fgfr4*, Sanger sequencing and subsequent ICE (Inference of CRISPR Edits) analysis with Synthego software (Hsiau et al., 2018). The efficacies of CRISPR-2 and−3 were assessed by amplifying a ~1 kb fragment around the prospective cut sites and performing the T7 Endonuclease assay (Guschin et al., 2010). For this, PCR products were denatured and re-annealed, and mismatches between re-annealed wildtype and CRISPR-edited sequences were detected by T7 Endonuclease I digest (NEB). Digests were visualized on 2% agarose gels. The following primers were used to produce PCR products containing the prospective cut sites:

**Table:** 

**sgRNA**	**TARGET SITE (5′-3′)**	**FORWARD PRIMER**	**REVERSE PRIMER**
*1*	*AGGAACG* *TTTGC* TGCCG GGAGGG	*CTGTAC* *TCC*GTAGA CTAGCC	*TGCTCT* *CTCA*CCTTG GAAAAA
*2*	AGTGTG GTTCCATC AGACCGTGG	*ACTGT* *CAAG*TTCCG CTGTCC	*ACAGG* *CATC*TCA CAGGCATT
*3*	TGCAGGGGA ATACACAT GTCTGG	*TTAAGAT* *GCG*TGTGT GAGCAC	*TGGAG* *AGTT*TGCT TGCTGTG

### Cardiac Looping

Stage 45 *Xenopus* tadpoles were paralyzed with benzocaine and scored under a stereomicroscope. Looping was determined by position of the outflow tract. D-loops were defined as outflow tracts directed to the right, and L-loops to the left.

### *In situ* Hybridization

Digoxigenin-labeled antisense probes for *pitx2* (TNeu083k20), *coco* (TEgg007d24), *xnr1* (TGas124h10), *gdf3* (Tgas137g21), *gsc* (TNeu077f20), *xnr3* (Tgas011k18), *foxj1* (Tneu058M03), *vent2* (BG885317), *myf5* (TGas127b01), *xbra* (TNeu024F07), *fgfr4* (Dharmacon, Clone ID: 7521919) were *in vitro* transcribed using T7 High Yield RNA Synthesis Kit (E2040S) from New England Biolabs. Embryos were collected at the desired stages, fixed in MEMFA for 1–2 h at room temperature (RT) and dehydrated in 100% ethanol. GRPs were dissected post fixation prior to dehydration. Briefly, whole mount *in situ* hybridization of digoxigenin-labeled antisense probes was performed overnight, the labeled embryos were then washed, incubated with anti-digoxigenin-AP Fab fragments (Roche 11093274910), and signal was detected using BM-purple (Roche 11442074001), as previously described in detail (Khokha et al., 2002).

### GRP Immunofluorescence

*Xenopus* embryos were collected at stage 17 and fixed for 2 h at RT in 4% paraformaldehyde/PBS. All samples were washed three times in PBS + 0.1% TritonX-100 (PBST) before incubating in PBST + 3% BSA blocking solution for 2 h at RT. Samples were then placed in blocking solution + primary antibody ON at 4°C. Samples were washed three times in PBST before incubating in blocking solution + secondary antibody/Phalloidin for 2 h at RT. Samples were washed three times in PBST and one time in PBS before mounting in Pro-Long Gold (Invitrogen) and imaging on a Zeiss 710 confocal microscope. Primary antibodies and dilutions for IF: Mouse monoclonal anti-acetylated tubulin, clone 6-11B-1, SIGMA Catalog: T-6793 (1:1,000); rabbit polyclonal anti-MYOD (aa1-150), LSBio, Catalog: LS-C143580 (1:200). Alexa488 and 594 conjugated anti-mouse and rabbit secondary antibodies were obtained from Thermo Fisher Scientific and used at a 1:500 dilution. Alexa647 phalloidin (Molecular Probes, 1:50) was used to stain cell boundaries.

### Quantifications

All quantifications were done using standard Student's *t*-tests and taking into account three or more replicate experiments. To measure GRP area, the GRP area was outlined based on cell morphology and measured in ImageJ. To measure the presomitic area of the GRP, the GRP was first outlined based on morphology and the myoD positive portion of this area was subsequently outlined and similarly measured. Cilia were counted by thresholding individual acetylated-tubulin immunostaining images in ImageJ and performing particle analysis. The cilia count was then additionally verified by manual counting. Embryo numbers: *N* = 15–25 for total GRP and PSM area and *N* = 10 for cilia numbers. Embryo numbers for phenotype quantification and *in situ* hybridization experiments are indicated in each figure.

## Results

### Fgfr4 Is Required for LR Development

**Figure 1:**
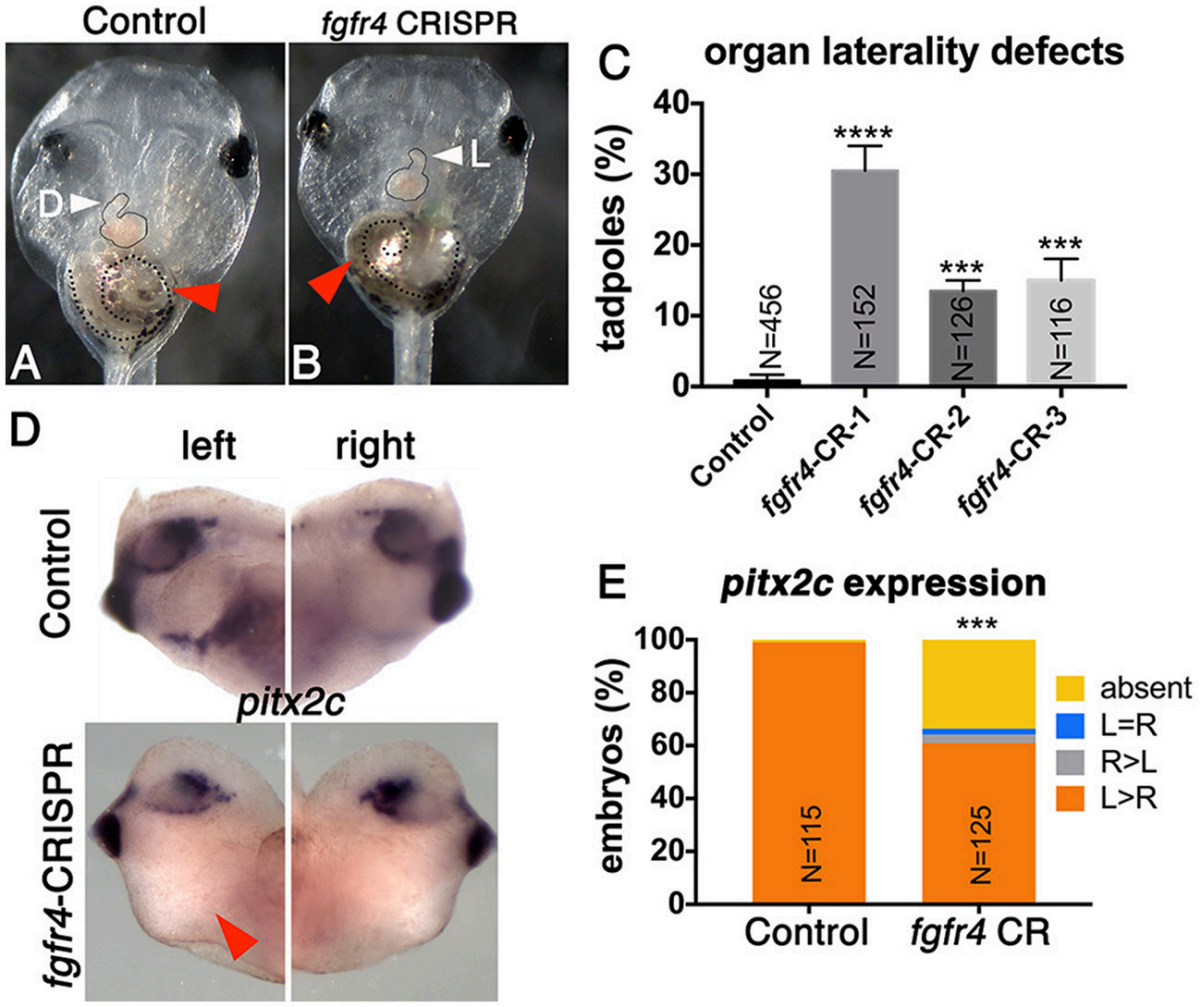
*Fgfr4* F0 CRISPR editing disrupts LR development. **(A,B)** *fgfr4* CRISPR tadpoles display organ laterality defects. Ventral view of a stage 45 live tadpole with a cardiac L-loop (**B**; outlined) and inverse gut coiling (**B**; dashed line and red arrowhead). **(C)** Percentages of tadpoles with laterality defects (L-loops and inverse gut coiling) for three different CRISPRs; tadpoles with L-loops and inverse gut coils were scored; only tadpoles with inverse but otherwise intact gut coiling were considered; animals with completely uncoiled guts were scored as normal, as this phenotype occurs in the control population as well. Tadpoles with both cardiac and gut looping defects were only counted once in this analysis. **(D)** *Pitx2c* expression in the LPM of tailbud stage animals (stage 28); red arrowhead indicates absent expression. **(E)** Percentages of stage 28 animals with different *pitx2c* phenotypes; ^****^p < 0.0001, ^***^p < 0.001.

To investigate whether *FGFR4* plays a role in LR development, we performed F0 CRISPR editing of the gene in *X. tropicalis*. This strategy effectively introduces damaging mutations in both alleles within 2 h after microinjection, and F0 tadpoles can be scored for organ situs 3 days later by simple inspection. We have found that known gene knockdown phenotypes can be replicated using F0 CRISPR editing in 9 out of 10 cases (Bhattacharya et al., 2015). In vertebrates, the cardiac tube initially forms in the midline, but then normally loops to the right (D-loop). Looping to the left (L-loop) or remaining midline (A-loop) is abnormal and suggests LR patterning defects consistent with heterotaxy (Baker et al., 2008; de Campos-Baptista et al., 2008; Rohr et al., 2008). Similarly, the gut depends on correct LR patterning to become coiled counter-clockwise and the intestinal rotation can be inverted in heterotaxy (Campione et al., 1999). We generated three sgRNAs independently targeting three non-overlapping sites in exons 3 (CRISPR-1), 6 (CRISPR-2), and 8 (CRISPR-3) of *fgfr4*, respectively. We verified gene modification using PCR amplification of the cut site followed by either Sanger sequencing and ICE (Inference of CRISPR Edits) analysis or the T7 Endonuclease I assay (Supplementary Figure 1). F0 CRISPR for all three sgRNAs led to tadpoles with cardiac L-loops and inverted gut looping (Figures 1A–C), indicating that *fgfr4* plays a role in establishing organ laterality.

Next, we tested if *fgfr4* depletion affected global LR patterning. Heart and gut looping both depend on activation of asymmetric gene expression in the left LPM prior to heart and gut tube morphogenesis (Logan et al., 1998; Piedra et al., 1998; Ryan et al., 1998; Yoshioka et al., 1998; Campione et al., 1999). Pitx2c, a homeobox transcription factor, is normally expressed in the left LPM. In a third of *fgfr4* CRISPR animals, we found *pitx2c* transcripts to be completely absent from the LPM, while a small minority displayed abnormal right-sided or bilateral *pitx2c* expression (Figures 1D,E). These results confirm that global LR development is compromised prior to organogenesis in *fgfr4*-depleted animals.

### *Fgfr4* Is Required for GRP Patterning

**Figure 2:**
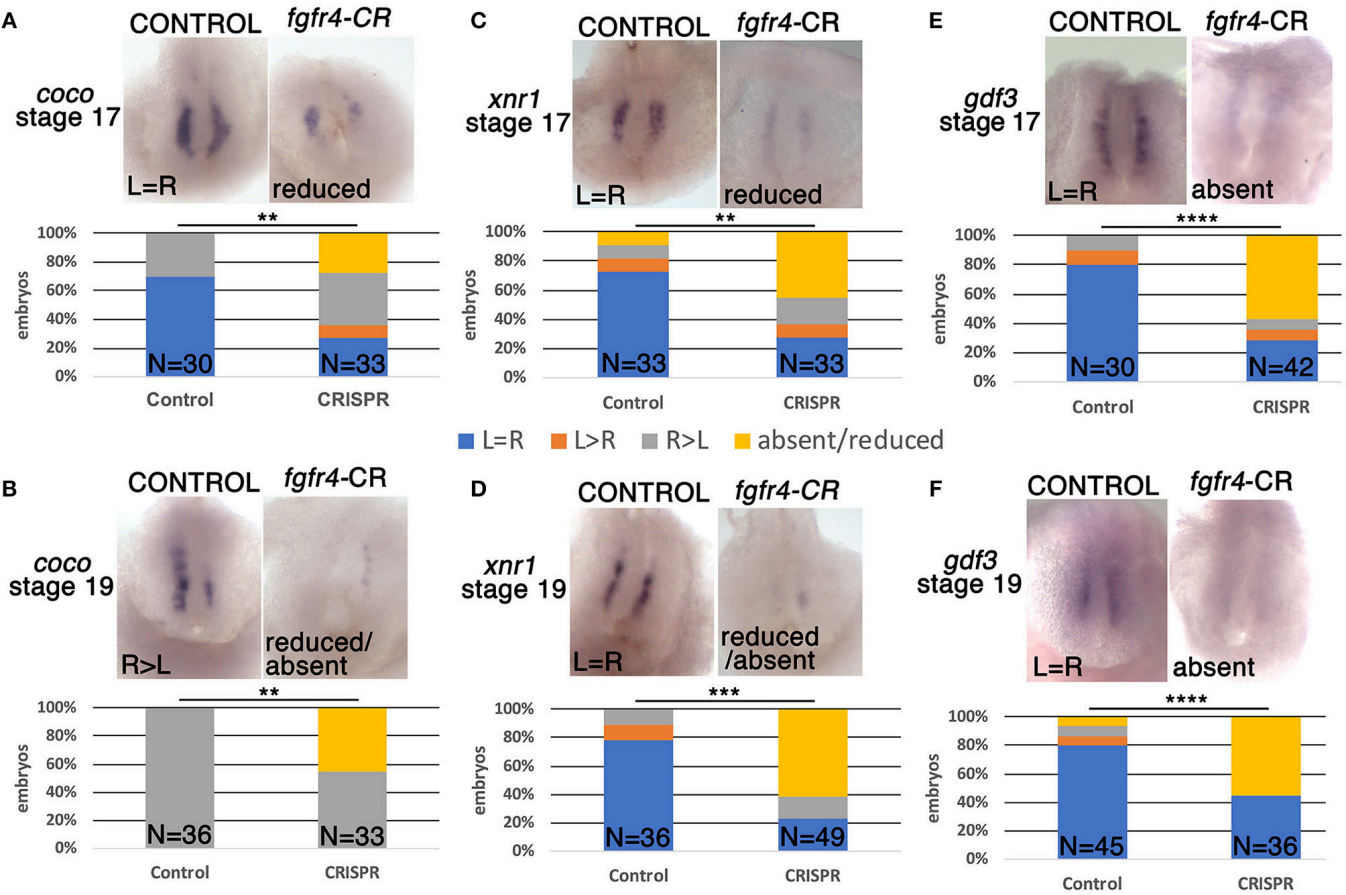
GRP patterning is defective in *fgfr4* CRISPR mutants. Most representative expression patterns of pre-somitic GRP markers *coco, xnr1*, and *gdf3* in stage 17 **(A,C,E)** and stage 19 **(B,D,F)**. GRPs of control and *fgfr4* F0 CRISPR animals; ventral view of GRPs, anterior is to the top. Graphs show percentages of embryos with differential expression patterns of *coco, xnr1*, and *gdf3*. ^**^p < 0.01, ^***^p < 0.001, ^****^p < 0.0001.

One of the first molecular targets to be asymmetrically expressed in the embryo upstream of *pitx2c* is the nodal antagonist *coco*. At the frog LR organizer (LRO), known as the GRP, *coco* expression is initially bilateral and then becomes downregulated on the left in response to leftward fluid flow generated by surface cilia (Schweickert et al., 2010). This allows downstream signaling via TGFbeta factors *xnr1* (nodal) and *gdf3* to take place exclusively on the left and become further transmitted to the left LPM (Vonica and Brivanlou, 2007), a cascade that is conserved among vertebrates (Nakamura and Hamada, 2012; Blum et al., 2014). To examine whether asymmetric gene expression was affected upstream of *pitx2c* at the LRO level, we examined *coco* expression at stage 19 after fluid flow in *fgfr4*-depleted embryos. Interestingly, we found *coco* to be bilaterally reduced or completely absent in almost half of the CRISPR embryos we analyzed (Figure 2B). We would predict that absence of *coco* would allow for bilateral TGFbeta signaling, resulting in bilateral *pitx2c* expression later in the LPM. Contrary to this scenario, we primarily encountered entirely absent *pitx2c* expression in *fgfr4* CRISPR animals. To understand this discrepancy, we examined the expression of TGFbeta factors *xnr1* and *gdf3* at the LRO. We found both transcripts, which normally have a predominantly bilateral expression, to be dramatically reduced or absent in over 50% of post-flow CRISPR embryos (Figures 2D,F). The absence of *xnr1* explains the complete bilateral lack of a left-handed signal and is consistent with absence of *pitx2c* expression. The reduction/absence of *gdf3* is also consistent with absent *pitx2c*, since *gdf3* facilitates the transmission of the nodal signal to the LPM (Vonica and Brivanlou, 2007).

To further investigate the loss of these lateral nodal-related signals, we considered the possibility that the GRPs of *fgfr4*-depleted embryos were fundamentally mispatterned even prior to fluid flow. At stage 16, before cilia driven flow is established, *coco, xnr1*, and *gdf3* transcripts are expressed mostly bilaterally in control embryos. Notably, *all* three transcripts were strongly reduced or absent (Figures 2A,C,E), indicating that the GRP is not patterned correctly in *fgfr4*-depleted embryos, irrespectively of fluid flow.

**Figure 3:**
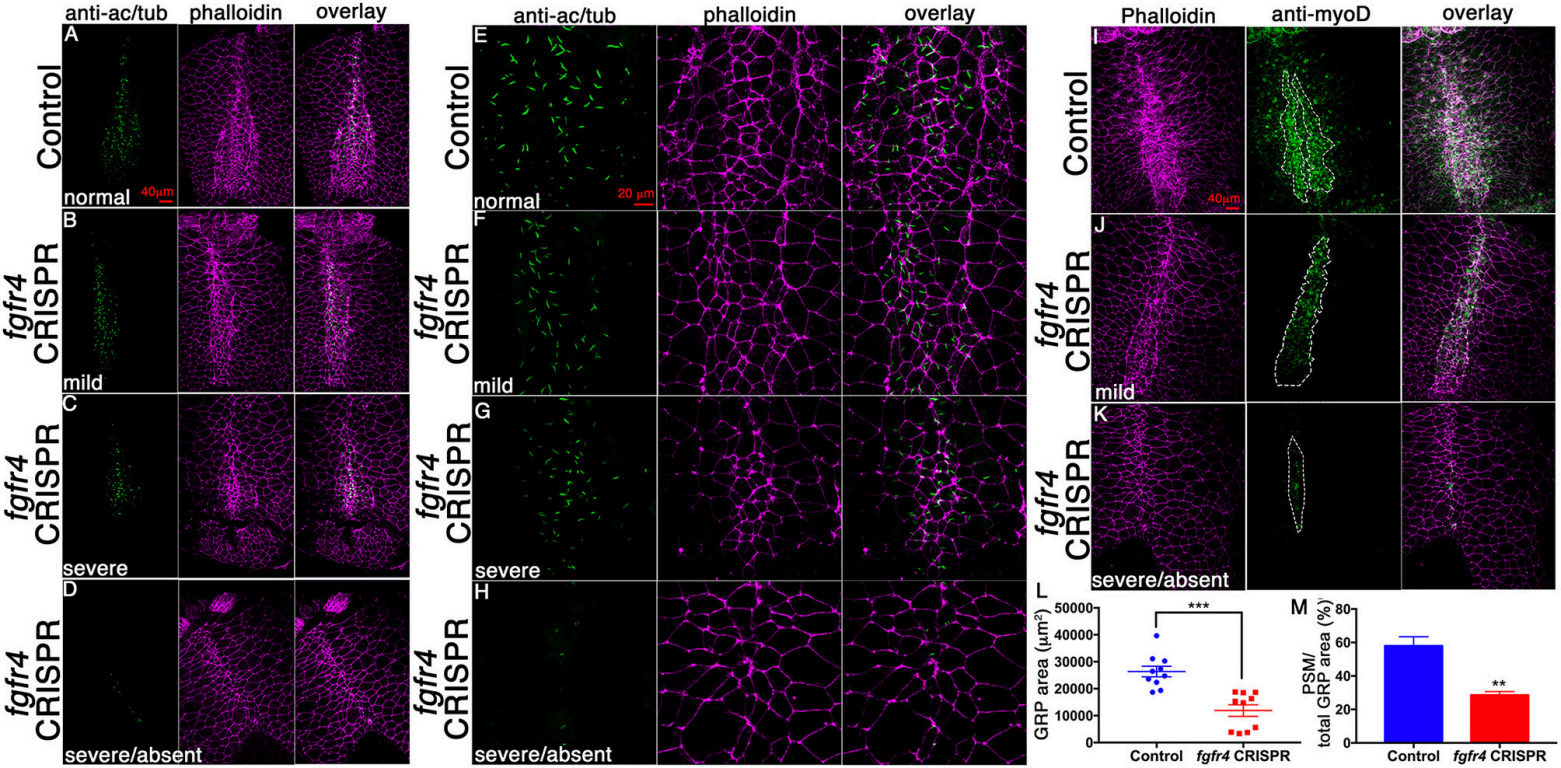
GRP morphology and identity are altered in *fgfr4* CRISPR embryos. **(A–D)** GRPs of *fgfr4* CRISPR animals are morphologically distinct, as shown by phalloidin (actin) and anti-acetylated tubulin (cilia) stain; phenotypes ranging from mild **(B)** to severe **(C,D)**, depending on loss of small mesodermal ciliated cells. **(E–H)** Higher magnification of GRPs shows loss of ciliated GRP area in *fgfr4* CRISPR embryos **(G, H)**. **(I–K)** The pre-somitic, myoD positive portion of the GRP (outlined) is drastically reduced in *fgfr4* CRISPR embryos, even in embryos in which the overall GRP morphology is preserved **(J)**. **(L)** Quantification of total GRP area, defined morphologically by small, ciliated cells, is reduced in *fgfr4* CRISPR embryos. **(M)** The myoD positive area of the GRP, normalized to total GRP area, is specifically reduced in *fgfr4* CRISPR embryos. Scale bars in **(A–D, I–K)** = 40 μm, in **(E–H)** = 20 μm. ^**^p < 0.01, ^***^p < 0.001.

Abnormal patterning of the GRP suggests that its cellular composition may be affected. To assess GRP morphology, we performed F-actin/phalloidin stain and acetylated tubulin immunostaining to visualize cell boundaries and cilia, respectively (Figures 3A–H). LRO cells of the GRP normally form a teardrop structure composed of small mesodermal ciliated cells. GRPs of *fgfr4* CRISPR embryos were morphologically distinct and composed of larger cells lacking cilia, resembling the neighboring endoderm (Figures 3A–D; phalloidin). Consistently, we measured a dramatically reduced total LRO area upon *fgfr4* knockdown (Figure 3L). In mildly affected GRPs, the natural teardrop shape of the LRO was preserved, albeit reduced in area (Figures 3B,F), whereas more severe cases displayed only one or two rows of mesodermal cells and lacked the regular teardrop structure (Figure 3D,H). Because pre-somitic GRP markers *coco, xnr1*, and *gdf3* were reduced or absent in *fgfr4* CRISPR embryos, we used an antibody against the myogenic transcription factor myoD to visualize the pre-somitic GRP (Figures 3I–K). The pre-somitic mesoderm (PSM) protrudes into the gastrocoel between the hypochordal central portion of the GRP and the surrounding endoderm. We found the myoD-positive portion of the GRP to be notably reduced relative to total GRP area in CRISPR embryos (Figures 3I–K,M), indicating a specific loss of pre-somitic GRP, which is consistent with the reduction in *coco, xnr1*, and *gdf3* expression. Finally, even though we counted fewer cilia per GRP in *fgfr4* CRISPR embryos (Supplementary Figure 2B), we quantified a similar cilia per GRP area ratio to that of control embryos (Supplementary Figures 2A,C), suggesting that *fgfr4* does not exert an effect on cilia differentiation *per se*, but rather affects the area of pre-somitic LRO. Altogether, this data suggests that *fgfr4* is required for pre-somitic GRP patterning.

### Fgfr4 Patterns the Paraxial Mesoderm During Gastrulation

**Figure 4:**
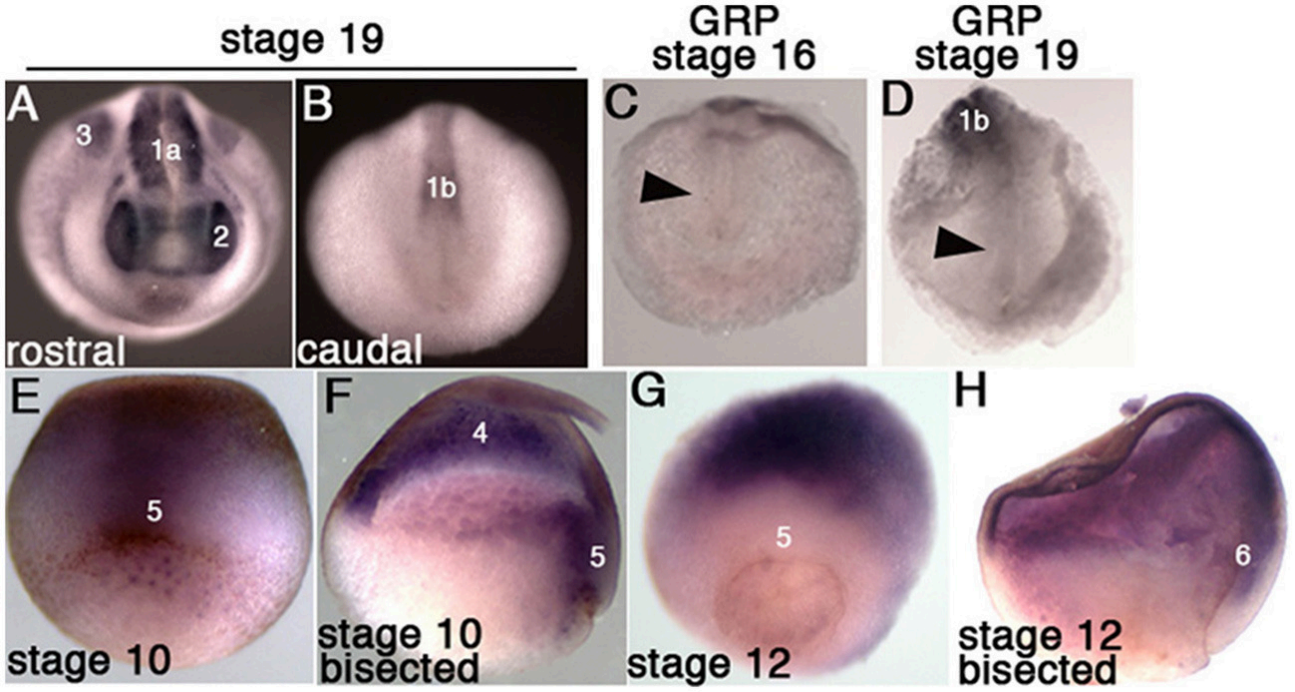
*Fgfr4* expression during early *X. tropicalis* development, detected by *in situ* hybridization. **(A,B)** Whole stage 19 embryos; expression is detected in the head region (2: eyes), lateral plate mesoderm (3), anterior somites (1a) and posterior pre-somitic mesoderm (1b). **(C,D)** Transcripts were not detected in stage 16 and 19 GRPs (arrowheads). **(E,F)** Stage 10.5 embryos, whole (**E**, dorsal view) or bisected through the dorsal midline **(F)**; *fgfr4* is broadly expressed in the ectoderm (4) and dorsal marginal zone (5). **(G,H)** Stage 12 embryos, whole (**G**, dorso-vegetal view) or bisected **(H)**; *fgfr4* is absent from the marginal zone (5), but is expressed in the anterior migrating involuted mesoderm (6).

**Figure 5:**
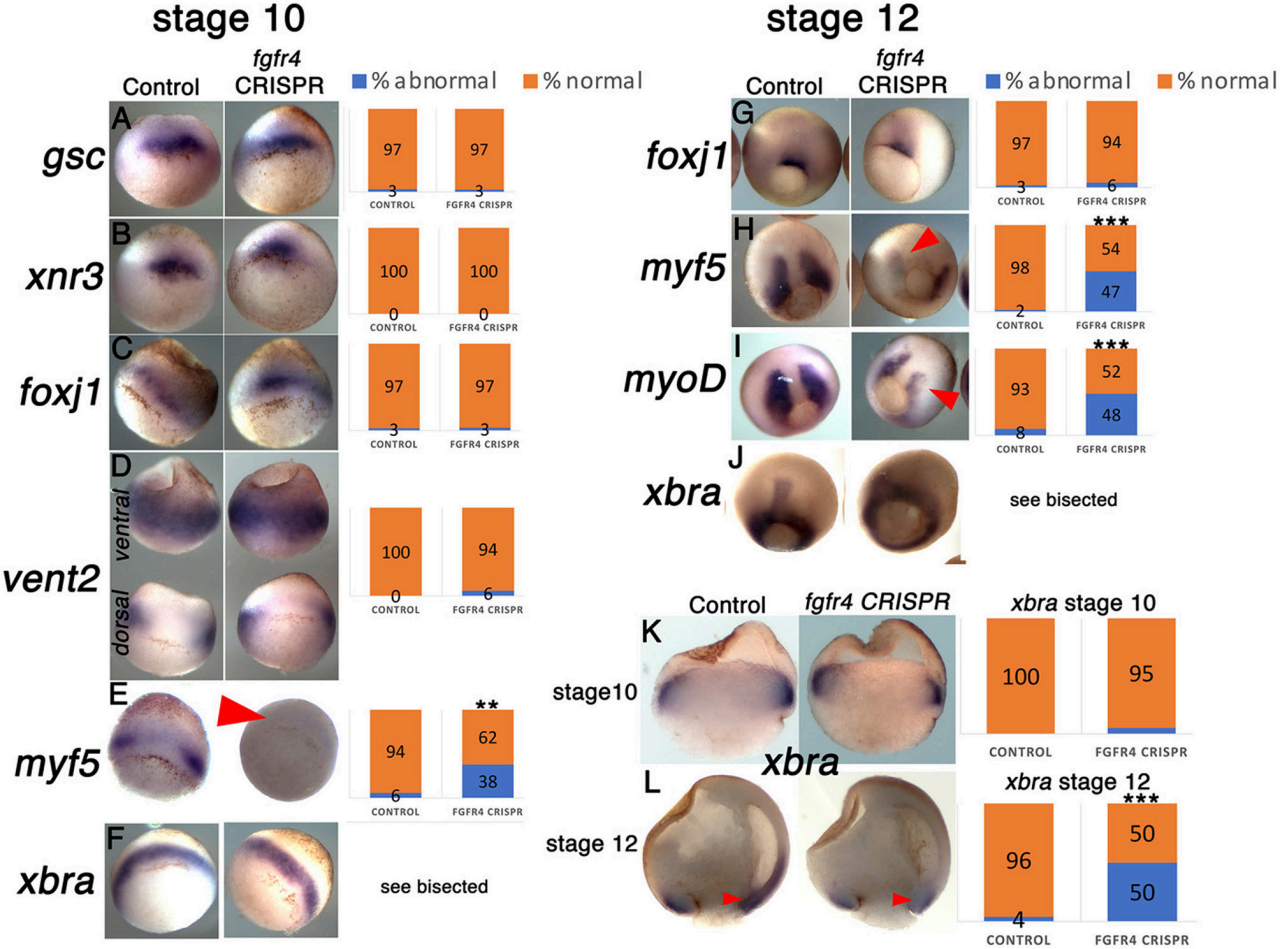
The paraxial myogenic mesoderm is mispatterned in *fgfr4* CRISPR embryos. **(A–F, G–J**) Dorsal-vegetal views showing expression of an array of mesodermal markers in stage 10 **(A–F)** and stage 12 **(G–J)** embryos. Expression of paraxial mesoderm markers *myf5* and *myoD* is perturbed in *fgfr4* CRISPR embryos. **(K, L)** *Xbra* expression in embryos bisected through their dorsal midline at stages 10.5 and 12; red arrowheads point at xbra expression in the involuted mesoderm. Graphs show percentages of embryos with normal vs. abnormal expression of each marker; *N* = 32–40 embryos per Control or *fgfr4* CRISPR; ^**^p < 0.001, ^***^p < 0.0001.

*Fgfr1* is expressed in the zebrafish LRO, where it activates essential ciliogenesis genes (Neugebauer et al., 2009). We considered that *fgfr4* may play a similar role in the GRP but were unable to detect *fgfr4* transcripts in the GRP (Figures 4C,D). Expression in the developing somites, eyes, and LPM of neurula stage embryos (Figures 4A,B,D) served as a positive control for transcript detection. The GRP is composed of hypochordal and PSM and both these tissues are specified during early gastrulation from dorsally located superficial (SM) and paraxial/myogenic mesoderm, respectively (Hopwood et al., 1989, 1991; Zetser et al., 2001; Shook et al., 2004; Stubbs et al., 2008; Walentek et al., 2013). Because *fgfr4* is expressed in the dorsal mesoderm during gastrulation (Figures 4E–H), we hypothesized that it could regulate early mesodermal patterning. In the early gastrula (stage 10), an array of markers is expressed in the mesoderm in a regionally restricted manner. We analyzed gene expression specific for the dorsal organizer (*gsc, xnr3*), paraxial/myogenic (*myf5*) mesoderm, superficial (*foxj1*) mesoderm, and ventral (*vent2*) mesoderm. Early gastrula (stage 10) *fgfr4*-depleted embryos showed intact patterning of the organizer, ventral, and superficial mesoderm but had absent *myf5* expression in the paraxial/myogenic mesoderm (Figures 5A–F). Moreover, the pan-mesodermal marker *xbra* was normally expressed throughout the mesoderm of stage 10 *fgfr4* CRISPR embryos, indicating that basic mesodermal identity was preserved even though *myf5* expression was absent (Figures 5F,K). Toward the end of gastrulation (stage 12), *myf5* expression was partially recovered, but markedly mispatterned (Figure 5H) and the upstream myogenic factor *myoD* was mispatterned in the same region (Figure 5I). The superficial mesoderm remained correctly patterned via *foxj1* at this stage (Figure 5G). Interestingly, midline bisection of late gastrula fgfr4-depleted embryos revealed an additional reduction in *xbra* expression in the involuted mesoderm (Figures 5J,L), confirming a previously reported relationship between FGF signaling and mesodermal *xbra* expression (Isaacs et al., 1994). These results altogether suggest that *fgfr4* is specifically required during gastrulation to pattern the paraxial/myogenic mesoderm and also maintain *xbra* expression in the involuted mesoderm. Loss of this paraxial mesoderm is then reflected in loss of lateral LRO markers *coco, xnr1*, and *gdf3* which results in LR patterning defects.

## Discussion

In this study, we propose a role for the candidate heterotaxy gene *FGFR4* in pattering the paraxial mesoderm, which contributes to the formation of the lateral LRO. The PSM of the GRP of *fgfr4* knockdown embryos lacks general myogenic patterning via *myoD*, but also *coco, xnr1*, and *gdf3*, which are specific markers for the PSM exposed to the gastrocoel and are indispensable for the GRP's function as a LRO (Vonica and Brivanlou, 2007; Schweickert et al., 2010).

Both the hypochordal and PSM, which compose the GRP, are specified during early gastrulation from superficial (SM) and paraxial mesoderm, respectively. Multiple genes are known to affect LR patterning via SM patterning, most of them via the ciliogenesis gene *foxj1* (Caron et al., 2012; Walentek et al., 2012; Griffin et al., 2018). In addition, knockdown of the global mesodermal determinant *Brachyury*/*xbra* results in LR defects both in frogs and mice (King et al., 1998; Kitaguchi et al., 2002). However, little is known about the signals that determine the fate of the PSM portion of the GRP during gastrulation, and while SM specification has been previously connected to candidate heterotaxy genes (Griffin et al., 2018), it is unclear whether PSM specification is similarly relevant. The PSM GRP is part of the greater paraxial mesoderm and thus also expresses myogenic markers during gastrulation (Shook et al., 2004; Schweickert et al., 2010). After gastrulation, the layer of PSM exposed to the gastrocoel becomes distinct from the more superficial PSM by additional patterning through factors (e.g., *coco, xnr1*, and *gdf3*) that facilitate its role as LRO tissue and enable the onset of asymmetric gene expression. At that stage, the PSM of the GRP still maintains the expression of myogenic factors like *myoD* (Schweickert et al., 2010). Given the key role of the PSM in the frog GRP, it is not surprising that paraxial mesoderm specification affects LR patterning. In mammals, the exact lineage of node cells and thus the contribution of paraxial mesoderm to the LRO remains to be determined. In mice, *Fgfr4* expression is detectable in paraxial myogenic mesoderm during node stages (Stark et al., 1991), and it would be interesting to examine whether its transcripts are present in the node region before LR cues become upregulated. Given that there are no reported LR phenotypes upon knockdown of the few known genes required for muscle development (Rudnicki et al., 1993; Pownall et al., 2002), it seems unlikely that the myogenic properties of paraxial mesoderm *per se* affect LRO function. It is rather likely that *fgfr4* controls an array of paraxial mesodermal genes during gastrulation, and that one or more of these genes are key to specify the GRP for LR cue expression.

FGF ligands FGF8 and FGF4 are required at several steps of LR development, which include LRO morphogenesis, ciliogenesis, and asymmetric gene expression at the LPM (Boettger et al., 1999; Meyers and Martin, 1999; Albertson and Yelick, 2005; Yamauchi et al., 2009). In addition, FGFR1 has been identified as essential for ciliogenesis once the LRO is shaped (Neugebauer et al., 2009). The role of FGFR4 appears distinctly different than that of FGFR1, since it acts to specify LRO tissue during gastrulation and is not expressed in the established LRO. We also observe fewer and sometimes shorter cilia in GRPs of *fgfr4* CRISPR embryos, but this effect is likely secondary to the patterning defect.

A long-standing connection exists between FGF signaling and gastrulation, and mesodermal patterning and morphogenesis in particular. Expression of dominant negative constructs for FGFR1 and FGFR4 effectively perturbs mesoderm induction by abolishing *xbra* expression (Amaya et al., 1991, 1993; Isaacs et al., 1994; Hardcastle et al., 2000). Moreover, depletion of ligand FGF4 (eFGF) strikingly resembles *fgfr4* knockdown by inhibiting *myoD* expression (Fisher et al., 2002) in the early mesoderm, suggesting that a fgf4/fgfr4 interaction may convey paraxial mesodermal specification during gastrulation.

Altogether our study establishes a link between FGFR4 and induction of paraxial mesoderm during gastrulation, which impinges on the specification of the pre-somitic GRP and its function as a LRO. These results help to construct the complex puzzle of FGF ligands and receptors that contribute to mesodermal and LR patterning in the early embryo.

## Author Contributions

ES performed GRP and gastrulation marker analyses, and manuscript writing. OL performed cardiac looping, *fgfr4* and *pitx2 in situ* hybridization analyses. SA collected embryos at various stages and contributed significantly to manuscript preparation. ES and MK conceived and planned experiments, and interpreted data.

### Conflict of Interest Statement

The authors declare that the research was conducted in the absence of any commercial or financial relationships that could be construed as a potential conflict of interest.

## Supplementary Material

Supplementary Figure 1 Injection of CRISPRs 1–3 results in edits in *fgfr4* in the genome of F0 frogs. **(A–C)** ICE (Inference of CRSIPR Edits) analysis of Sanger sequencing data from a genomic 800 bp PCR-fragment that contains the target site for CRISPR-1. **(A)** Average editing and knock-out scores for CRISPR-1 as identified by the ICE/Synthego software (*N* = 8 for control/*N* = 8 for CRISPR). All eight CRISPR F0 tadpoles displayed edits at the *fgfr4* target cut site, with overall editing efficiency within a single tadpole ranging from 75 to 95%, and knockout efficiency from 27 to 61%. In contrast, none of the Control tadpoles displayed mutations in the same genomic region. **(B)** Inferred distribution of Indels around the *fgfr4* CRISPR-1 target site within a single F0 CRISPR animal. The x-axis indicates the size of the insertion/deletion and the y-axis shows the percentage of sequences that contained it. **(C)** Relative contributions of inferred sequences present in a single CRISPR-1 F0 animal. An example for one single animal is shown. The cut site is presented with a black vertical dotted line and the wildtype sequence is marked by a “+” symbol on the far left. **(D)** T7 Endonuclease assay for *fgfr4* CRISPRs-2 (up) and−3 (down). Lanes show PCR products that contain the prospective cut sites, amplified from genomic DNA of different animals (numbered; *N* = 3–4 per CRISPR, *N* = 2 per Control), prior to digestion with T7 Endonuclease I (a) and after digestion (b). Red arrowheads point at fragments that are unique in CRISPR animals post-digestion and correspond to predicted fragment sizes: 300/550 bp for CRISPR-2 and 400/600 bp for CRISPR-3.

Supplementary Figure 2 Cilia number in the GRP of *fgfr4* CRISPR-1 F0 embryos. **(A)** An example of how GRPs were outlined in order to count cilia and measure GRP area. **(B,C)** The total number of cilia per GRP is reduced in *fgfr4* CRISPR embryos **(B)**, however when the cilia numbers are normalized to total GRP area, no difference is visible between control and CRISPR embryos **(C)**. Scale bar = 40 μm.
