# Supplementary figures and images for: Candidate Heterotaxy Gene FGFR4 Is Essential for Patterning of the Left-Right Organizer in Xenopus
Source: Front Physiol. 2018 Dec 4;9:1705. doi: 10.3389/fphys.2018.01705 (PMC6288790; doi:10.3389/fphys.2018.01705)

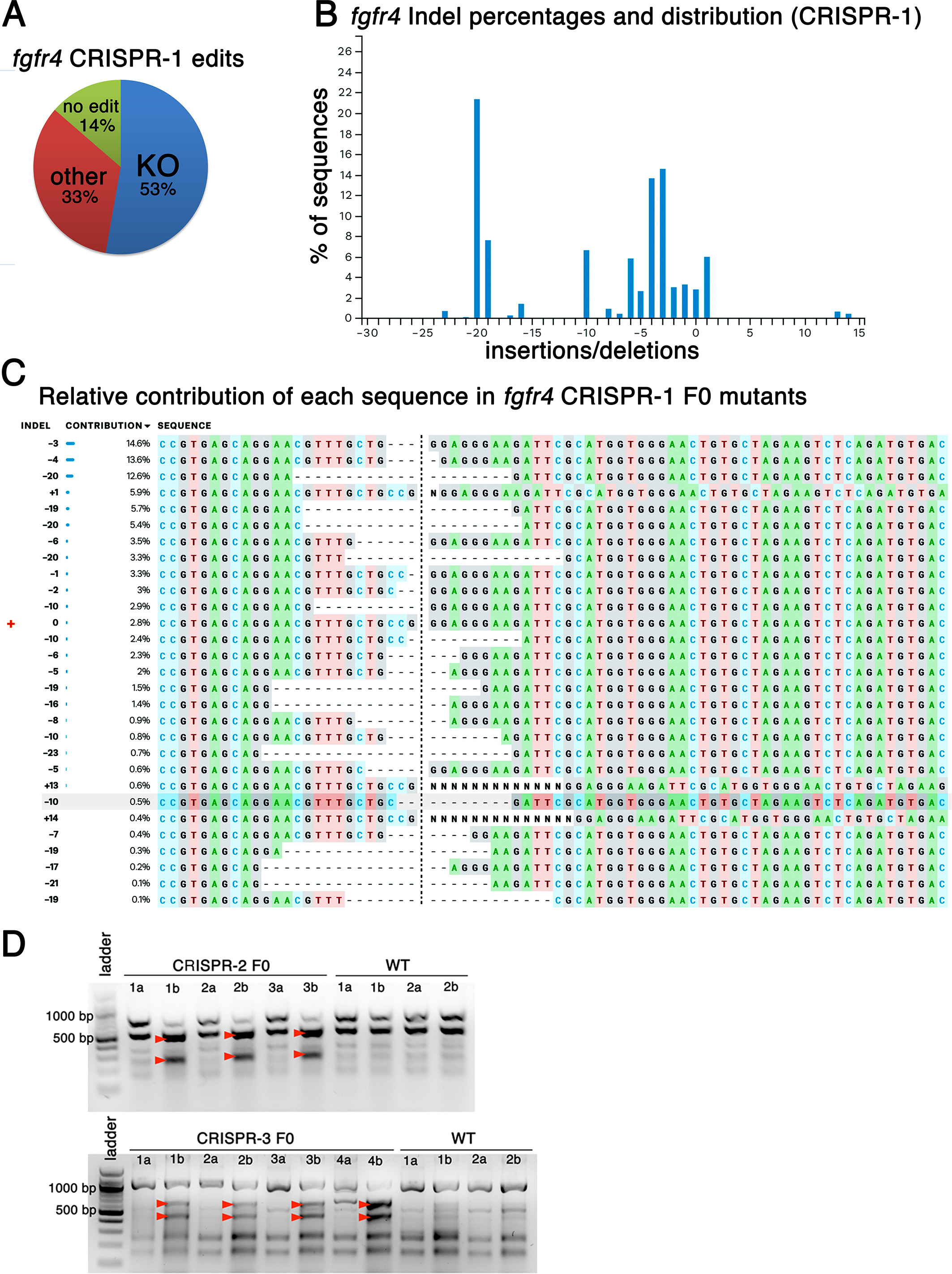

Supplement: Supplementary Figure 1 — Injection of CRISPRs 1–3 results in edits in fgfr4 in the genome of F0 frogs. (A–C) ICE (Inference of CRSIPR Edits) analysis of Sanger sequencing data from a genomic 800 bp PCR-fragment that contains the target site for CRISPR-1. (A) Average editing and knock-out scores for CRISPR-1 as identified by the ICE/Synthego software (N = 8 for control/N = 8 for CRISPR). All eight CRISPR F0 tadpoles displayed edits at the fgfr4 target cut site, with overall editing efficiency within a single tadpole ranging from 75 to 95%, and knockout efficiency from 27 to 61%. In contrast, none of the Control tadpoles displayed mutations in the same genomic region. (B) Inferred distribution of Indels around the fgfr4 CRISPR-1 target site within a single F0 CRISPR animal. The x-axis indicates the size of the insertion/deletion and the y-axis shows the percentage of sequences that contained it. (C) Relative contributions of inferred sequences present in a single CRISPR-1 F0 animal. An example for one single animal is shown. The cut site is presented with a black vertical dotted line and the wildtype sequence is marked by a “+” symbol on the far left. (D) T7 Endonuclease assay for fgfr4 CRISPRs-2 (up) and−3 (down). Lanes show PCR products that contain the prospective cut sites, amplified from genomic DNA of different animals (numbered; N = 3–4 per CRISPR, N = 2 per Control), prior to digestion with T7 Endonuclease I (a) and after digestion (b). Red arrowheads point at fragments that are unique in CRISPR animals post-digestion and correspond to predicted fragment sizes: 300/550 bp for CRISPR-2 and 400/600 bp for CRISPR-3. [file Image_1.JPEG]

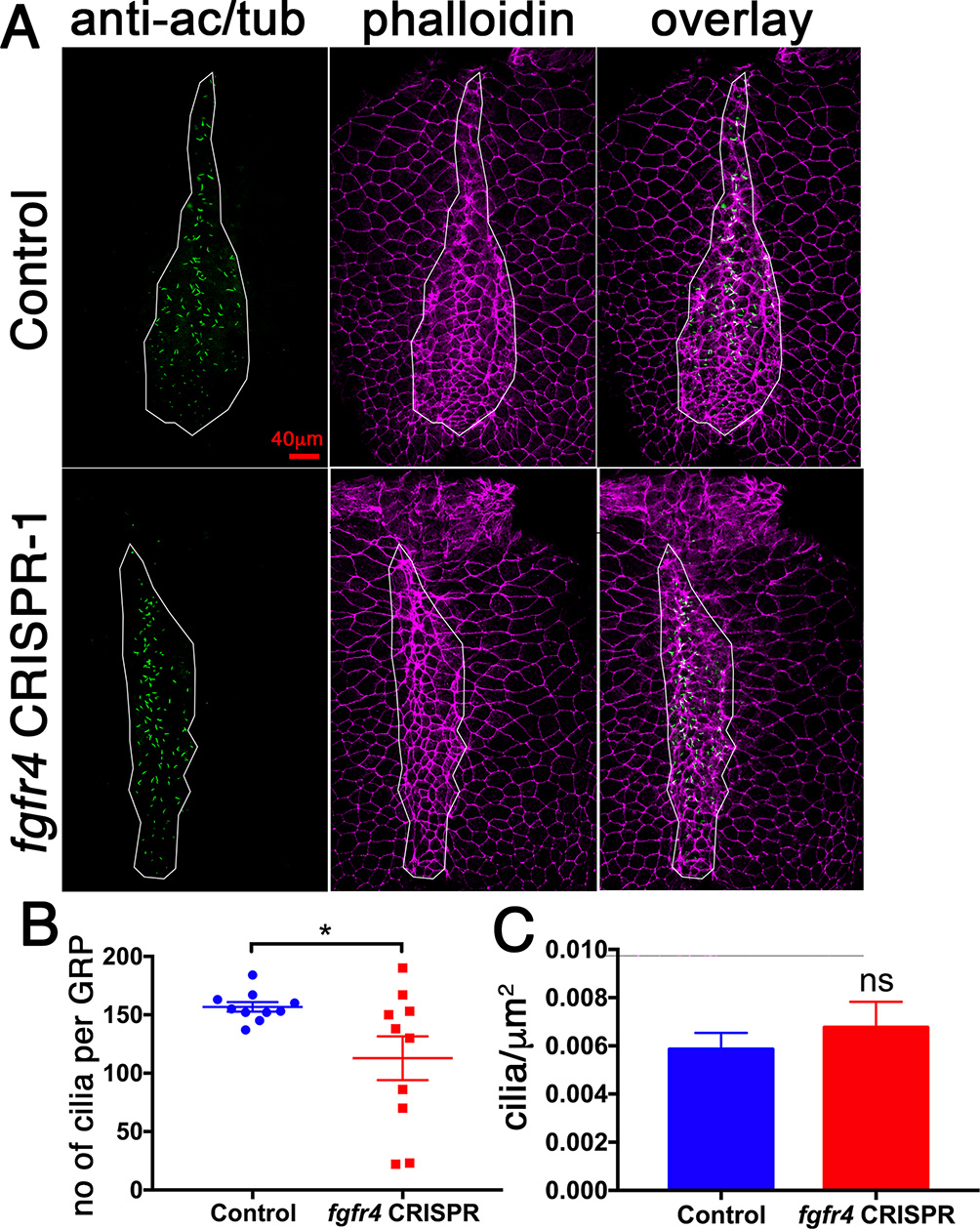

Supplement: Supplementary Figure 2 — Cilia number in the GRP of fgfr4 CRISPR-1 F0 embryos. (A) An example of how GRPs were outlined in order to count cilia and measure GRP area. (B,C) The total number of cilia per GRP is reduced in fgfr4 CRISPR embryos (B), however when the cilia numbers are normalized to total GRP area, no difference is visible between control and CRISPR embryos (C). Scale bar = 40 μm. [file Image_2.JPEG]
